# Roswell Park Medical Club

**Published:** 1901-12

**Authors:** George F. Cott

**Affiliations:** Secretary


### Roswell Park Medical Club.

REPORTED BY GEORGE F. COTT, M. D., Secretary.

The second meeting of the Roswell Park Medical Club, was held October 7, 1901, at the club rooms in the Mooney-Brisbane Building.

The paper of the evening was read by Dr. J. H. Potter, entitled, Consideration of some of the functional signs of diseases of the stomach. (See page 341.)

DISCUSSION.

Dr. BAGLEY—This is a good paper, but a little deep. I think a whole lot depends upon familiarity with the method in use for examining stomach contents, which Dr. Potter laid before us tonight. It is a question whether the general practitioner has the time to go into it. A specialist, of course, knows much more about it. The absence of HCl. may mean cancer and it may not.

Dr. MEISBURGER called attention to Einhorn's stomach bucket, preferring it to the more uncomfortable tube.

Dr. THOMPSON—I was much interested in the paper of the evening and agree with Dr. Potter in the importance of working out carefully cases of so-called indigestion. There can be nothing more unscientific than the old way which may still obtain at times of calling certain cases of dyspepsia, and proceeding to administer drugs without knowing there is the least scientific indication for their use. Some years ago I learned from Dr. Stockton his methods of securing and analysing stomach contents, and in all important cases coming to me, I use more or less the methods outlined by the essayist. The device exhibited by Dr. Potter for securing stomach contents undiluted is new to me, and I am thankful to have seen it. I will certainly use it in the future. I would add that after testing for hydrochloric acid it is not often necessary to test for the fatty acids; if present, they can usually be detected by the sense of smell.

Dr. Geo. F. Cott read a paper on the Non-surgical treatment of some of the commonest forms of deafness. (See page 349.)

DISCUSSION.

Dr. Gibson—I think the essayist, heading the paper as he does, Treatment of the commonest forms of deafness, attributes too much to the general practitioner and I think such cases should be relegated to the specialist unless he directs the proper course of treatment. My own experience is of no particular value.

Dr. Bagley—We cannot expect too much of the general practitioner. Such cases are very stubborn and though we occasionally get good results, it is often better to send them for special treatment. Last winter I treated one case with the politzer bag, which improved materially, still the discharge continued after the treatment was stopped.

Dr. Meisburger confined his treatment of ear diseases to those cases of deafness caused by incipated cerumen or a neglected plug of cotton.

Dr. J. H. Potter highly commended the paper as plain, practical common sense, and, though coming from a specialist, such as the general practitioner can understand. One of the chief difficulties was to keep the patient under control long enough to accomplish any satisfactory result. In this respect the specialist has a great advantage.

Dr. McIntyre commended Dr. Gibson's view that such cases should be treated by the specialist or under his direction. The matter of accuracy of deafness is of the utmost importance.

Dr. Thompson, as president, tendered the thanks of the club for the essay.

Dr. Cott said, in closing the discussion, that his purpose in writing the paper had been to bring in the subject before the general practitioner. It had never been presented in this manner by any text-book at their disposal. None of them go into detail. He has very little difficulty in controlling patients and holding them for any length of time. They are always informed that it may require from three months to three years to gain permanent result. A certain small percentage of cases can not be improved under any kind of treatment and a few even get worse. Differential diagnosis is not surrounded by as many difficulties as it appears. Cases resolve themselves into two great classes: hypertrophy and atrophy, or sclerosis. A vast majority of cases, over 80 per cent., occur in the young. In sclerosis we have a thining of the membrane and connective tissue formation, and then again treatment in hypertrophy gives always immediate result, if but momentary. In sclerosis, treatment makes absolutely no impression and that is one of the cardinal points in diagnosis. When deafness is due to obstruction in the external meatus, the history is generally that of sudden onset. It is remarkable how much can be heard through a pinhole opening, an epithelial scale or other foreign substance shutting off this opening, suddenly cuts off the sound waves and thus deafness follows. Another condition of sudden deafness may occur in a case of apoplexy during sleep. This must be differentiated from deafness caused by cerumen. In order to treat deafness successfully, a normal nose and post nasal space is necessary. After having accomplished that, the deafness becomes stationary for quite a while, but by proper treatment, gradually improves. In many cases we have a condition of hyperplasia instead of organised hypertrophy, and which gradually subsides by equalising air-pressure and a more or less normal condition ensues.

